# Laparoscopic partial nephrectomy: Newer trends

**DOI:** 10.4103/0970-1591.57931

**Published:** 2009

**Authors:** Monish Aron, Burak Turna

**Affiliations:** Center for Laparoscopic and Robotic Surgery, USC Institute of Urology, Keck School of Medicine, University of Southern California, Los Angeles, CA, USA; 1 Department and Ege University, Izmir, Turkey

**Keywords:** Nephron Sparing Surgery, RCC, laparoscopy

## Abstract

**Objectives:**

To report the advances in laparoscopic partial nephrectomy (LPN) for renal masses with emphasis on technically challenging cases.

**Methods:**

Literature in the English language was reviewed using the National Library of Medicine database using the key words kidney, renal, tumor, nephron sparing surgery, and laparoscopic partial nephrectomy, for the period between 1993 and 2009. Over 500 articles were identified. A total of 50 articles were selected for this review based on their relevance to the evolution of the technique and outcomes, as well as expanding indications for LPN.

**Results:**

In expert hands, LPN is safe and effective for central tumors, completely intrarenal tumors, hilar tumors, tumor in a solitary kidney, large tumors requiring heminephrectomy, cystic tumors, multiple tumors, obese patients, and even incidental stage ≥ pT2 tumors. Perioperative outcomes and 5-year oncologic outcomes after LPN are comparable to open partial nephrectomy (OPN).

**Conclusions:**

In experienced hands indications for LPN have expanded significantly. In 2009, advanced LPN remains a skill-intensive procedure that can nevertheless provide excellent outcomes for patients with renal tumors.

## INTRODUCTION

Current literature supports nephron sparing surgery (NSS) in patients with a small renal mass (SRM) in the presence of a normal contralateral kidney (elective partial nephrectomy).[1–2] This is based on the fact that patients at risk of developing chronic kidney disease (CKD) are likely to benefit from the functional advantage of NSS. In addition, a significant fraction of SRMs presumed malignant are proven benign on final histopathology.[3–4]

Survival and recurrence rates with partial and radical nephrectomy for SRMs (≤ 4 cm) have been shown to be comparable.[5–6] However, radical nephrectomy has been shown to be an independent predictor for the development of CKD and hence should not really be the gold standard for treatment of SRM.[7] Initially, elective partial nephrectomy was reserved for renal tumors ≤ 4cm (T1a), however, recent data indicate that functional and oncological outcomes are similar even for selected tumors 4-7 cm (T1b) in size.[8–10]

The dissemination of laparoscopic and robotic techniques over the last decade has made it possible for partial nephrectomy for most small renal tumors to be performed in a minimally invasive fashion, with functional and oncological outcomes being comparable to open partial nephrectomy (OPN).[11–12]

Nevertheless, laparoscopic partial nephrectomy (LPN) is technically demanding. Secure renal hemostasis is the main challenge. Urine leak is a potential complication, prevention of which mandates meticulous suture closure of the collecting system. With increasing expertise and experience post-operative bleeding and urine leak rates have decreased substantially.[13] At the same time, indications for LPN are increasing to include more challenging cases: central tumors infiltrating into the renal sinus, completely intrarenal tumors, hilar tumors, tumor in a solitary kidney, large tumors requiring heminephrectomy and multiple tumors.[14]

This review discusses the evolution of the technique and expanding indications for LPN and attempts to provide specific tips and caveats for the successful performance of advanced LPN.

## TECHNICAL ASPECTS

Winfield *et al.* reported the first transperitoneal LPN in 1993.[15] A year later, Gill *et al.* described the technique of retroperitoneal LPN.[16] In the early days, LPN was reserved for small, solitary, exophytic, and peripheral tumors.[17,18] Recent refinements in techniques and technology have enabled the application of LPN to more complex tumors.

The principal technical challenge during LPN stems from the complexity of laparoscopic tumor excision and renal reconstruction in a time-sensitive manner. LPN for complex tumors requires an in-depth understanding of 3-D renal anatomy, an appreciation of visual cues during laparoscopy, as well as masterful ambidextrous laparoscopic suturing.

## TRANSPERITONEAL OR RETROPERITONEAL ACCESS

A surgeon who performs advanced LPN needs to be facile with both retroperitoneal and transperitoneal laparoscopy. The transperitoneal approach offers a larger working space, more familiar landmarks, and technical ease of suturing. While it is true that most tumors can be treated with transperitoneal LPN, there are some tumor locations that are easier to treat retroperitoneally.

As such, the choice of approach is dictated primarily by surgeon experience and tumor location. Other factors include tumor size, number of tumors, number of arteries supplying the kidney, amount of visceral fat surrounding the kidney, and route of any prior open surgery on the quadrant of interest.

Wright and Porter compared 32 retroperitoneal with 19 transperitoneal LPN.[19] The choice of approach was based on tumor location. The retroperitoneal approach was associated with shorter operating time, decreased blood loss, quicker return of bowel function and shorter hospitalization. They preferred the retroperitoneal approach for polar and posterolateral masses and transperitoneal approach for anterior and medial lesions.

We retrospectively compared 100 transperitoneal with 63 retroperitoneal LPN.[20] In our series, blood loss, perioperative complications, postoperative serum creatinine, analgesic requirements, and histological outcomes were comparable in the two groups.

Currently, we prefer the transperitoneal approach for all renal tumors except those that are located posteriorly or posteromedially on the upper pole. Angles for suturing in these locations are optimal with retroperitoneal access.

## RENAL SINUS AND PARENCHYMAL HEMOSTASIS

Achieving hemostasis in the partial nephrectomy bed is the most important challenge during LPN. Several strategies have been employed for this purpose. These include suture repair, use of biological hemostatics, radiofrequency ablation prior to LPN, laser dissection, waterjet dissection, and microwave tissue coagulation. A number of biologic hemostatics and sealants are commercially available: gelatin matrix thrombin sealant (Floseal^^®^^, Baxter, Deerfield, IL), fibrin glue (Tisseel^®^, Baxter), polyethylene glycol hydrogel (Coseal^®^, Baxter), cyanoacrylate glue (Dermabond^®^, Ethicon, Somerville, NJ), and Bioglue^®^ (CryoLife, Inc., Atlanta, GA).

Our preference is for conventional sutured renal reconstruction using FloSeal^®^ and a Surgicel bolster as hemostatic adjuncts. We compared our results in patients undergoing LPN with adjunctive use of Floseal with patients who underwent LPN without Floseal.[13] The Floseal group was associated with decreased hemorrhagic complications (12% vs. 3%) and significantly decreased overall complications (37% vs. 16%).

Herrell and Levin evaluated the TissueLink (TissueLink Medical, Inc., Dover, Delaware) radiofrequency device during unclamped LPN in the laboratory and in 25 human cases. Fibrin glue was used as a hemostatic adjunct. There were no intraoperative complications. Mean estimated blood loss was 98 ml (range 10-337). This device could potentially have a role in unclamped LPN for small, peripheral and exophytic tumors.[21]

Two types of lasers have been used for LPN on animals. Moinzadeh *et al.* performed 12 LPN using an 80 W potassium-titanyl-phosphate laser (KTP) (GreenLight PVP, Laserscope, San Jose, CA) without hilar clamping in the calf model.[22] This initial study of laparoscopic KTP laser partial nephrectomy without hilar clamping confirmed its technical feasibility in most cases and good short-term outcomes. Lotan *et al.* utilized a holmium:yttrium aluminum garnet (Ho:YAG) laser in 10 porcine kidneys to transect the lower pole followed by placement of fibrin glue on the cut surface.[23] Blood loss was minimal, but extravasation was noted on retrograde pyelogram in 2 animals in the survival group. The use of lasers for LPN appears promising although clinical data are awaited.

Moinzadeh *et al.* evaluated water-jet assisted (Helix Hydro-jet, Erbe-USA, Marietta, GA) LPN without renal hilar control in the survival calf model.[24] They were able to perform 18 of 20 cases without hilar control, with an estimated blood loss of 60 ml. Pelvicaliceal suture repair was necessary in 5 of 10 chronic kidneys, but no animal developed a urinary leak.

Till such time that one of these methods is clinically proven to be safe and effective in terms of hemostasis and pelvicaliceal repair, time tested techniques of sutured reconstruction will remain the reference standard.

## HILAR CLAMPING AND WARM ISCHEMIA

Except for the most superficial and exophytic tumors, most authors agree that substantial LPN requires a certain period of hilar clamping. Hilar clamping allows precise tumor excision and renal reconstruction in a near-bloodless field. Guilloneau *et al.* compared 12 patients undergoing LPN with hilar clamping vs. 16 patients without clamping (ultrasonic shears and bipolar cautery).[25] Clamping the renal vessels was associated with decreased blood loss and shorter laparoscopic operating room (OR) time.

Shekarriz *et al.* assessed the impact of warm ischemia on renal function in 17 patients undergoing LPN. Authors reported that in patients with contralaterally functioning kidney, temporary hilar clamping with a mean (warm ischemia time) WIT of 22.5 minutes resulted in preservation of renal function in the affected kidney.[26]

The limit of safe renal warm ischemia time has generally been considered to be 30 minutes. This limit was derived from canine studies performed over two decades ago. Clearly, this question needs to be revisited in a more scientifically rigorous manner. Till the time this issue is better understood, all efforts should be made to keep warm ischemia time to a minimum, especially since recent data indicate that 20 minutes may be a superior cut-off limit for renal warm ischemia.[27] Recent technical modifications have already allowed reduction of warm ischemia time during LPN to approximately 15 minutes in the majority of cases using the early unclamping technique.[28]

## LOCAL RENAL HYPOTHERMIA

Three main techniques for laparoscopic renal hypothermia exist: surface cooling with ice slush, instillation of cold saline through a retrograde ureteral catheter, and intra-arterial perfusion of cold saline.[29–31] Although these techniques are technically feasible and somewhat effective, they are rarely employed during clinical LPN. This is not just due to their complexity, but also because the majority of tumors treated with LPN do not require an unusually prolonged period of warm ischemia. Having said that, there is no doubt that a safe, effective, reproducible and user-friendly technique of laparoscopic renal hypothermia is likely to expand the indication for LPN further to include complex central tumors requiring delicate intra-renal reconstruction.

## COLLECTING SYSTEM REPAIR

Tumors abutting the collecting system requiring entry into the pelvicaliceal system (PCS) during excision are very common in our practice. We routinely employ a 5F ureteral catheter placed transurethrally in the renal collecting system to help identify PCS entry. Desai *et al.* prospectively compared the perioperative outcomes of 27 LPN with pelvicaliceal entry with 37 LPN with no pelvicaliceal entry.[32] Both groups were comparable in terms of OR time, tumor excision time and EBL. However, pelvicaliceal repair was associated with a longer WIT and hospital stay. None of the patients undergoing pelvicaliceal suture repair developed a urinary leak. The results of this study showed that intentional entry into the pelvicaliceal system for invasive tumors could be safely and effectively repaired. We currently suture repair the PCS with a running 3-0 polyglactin suture, and test the integrity of the repair with a retrograde injection of dilute methylene blue.

## INTRAOPERATIVE ULTRASOUND

The use of intraoperative ultrasound (US) has been advocated to facilitate advanced laparoscopic surgery. In our experience, expertly performed real-time ultrasonographic delineation of the tumor is extremely useful to plan resection during LPN, especially for non-exophytic tumors. Fazio *et al.* nicely showed that intraoperative US was very useful in advanced laparoscopic surgeries including LPN, laparoscopic radical nephrectomy, laparoscopic renal cryoablation (LRC), retroperitoneal exploration and resection of renal artery aneurysm.[33]

## EXPANDING INDICATIONS

With increasing laparoscopic confidence and experience many surgeons have attempted to expand the frontiers of laparoscopic renal surgery to include technically challenging cases. Specific to LPN, these carefully expanded indications are: 1) concomitant en bloc adrenalectomy, 2) presence of renal artery disease, 3) anomalous kidneys, 4) multiple tumors, 5) large tumors requiring heminephrectomy, 6) cystic tumors, 7) hilar tumors, 8) tumor in a solitary kidney, 9) central tumors, 10) ≥ pT2 tumors, 11) obese patients, and 12) ipsilateral prior renal surgery.[34]

## CONCOMITANT ADRENALECTOMY

From a technical standpoint, an upper pole tumor involving the adrenal gland may require LPN and concomitant adrenalectomy. Ramani *et al.* published their results in 4 patients undergoing transperitoneal LPN and concomitant adrenalectomy for upper pole tumor with suspected adrenal involvement. All patients were free of disease with a mean followup of 6.2 months.[35] The adrenal was maintained en bloc with the partial nephrectomy specimen and the overlying fat and fascia. This requires dividing the adrenal vessels first and completely mobilizing the upper pole of the kidney and adrenal outside Gerota's fascia prior to hilar clamping and LPN.

## CO-EXISTING RENAL ARTERY DISEASE

Renal artery disease coexisting with RCC presents unique management issues. Precise atraumatic dissection of the renal arterial branches is an advanced laparoscopic maneuver. Steinberg *et al.* described the technical considerations of LPN in 2 complicated cases involving kidneys with renal arterial disease.[36] The use of intra-operative Doppler ultrasound in the hands of an expert sonologist provided detailed information about the renal artery and its branches. Pre-operative 3-D CT is critical for surgical planning as it clearly shows location of arterial plaques and stents. Control of individual renal arterial branches with bulldog clamps may be required in situations where there is a stent or a plaque in the main renal artery. Direct application of a clamp over an area of plaque can lead to plaque rupture and aneurysm. At the completion of the LPN, if the kidney does not pink-up evenly after hilar unclamping, intra-operative Doppler ultrasonography should be performed.

## HORSE-SHOE KIDNEY

Horseshoe kidney is one of the most common renal anomalies. Molina *et al.* reported the initial case of LPN in a horseshoe kidney for a 2-cm complex cystic renal mass in the right moiety.[37] Posterolateral location of the renal mass prompted the authors to approach the tumor retroperitoneoscopically. For anomalous kidneys, detailed pre-operative radiological evaluation of the renal vasculature using 3-D reconstruction of triphasic CT is necessary for surgical planning. The surgical approach depends primarily on the location of the tumor. Both types of vascular clamps, Satinsky, and bulldogs need to be available on the instrument table.

## MULTIPLE TUMORS

Although radical nephrectomy is the gold standard in the presence of ipsilateral multiple tumors, NSS should be strongly considered in patients with decreased renal reserve. Steinberg *et al.* published their results in 13 patients (with an imperative indication in 92%) undergoing laparoscopic NSS for two or more ipsilateral renal tumors.[38] LPN was performed in 6 patients either alone or in combination with laparoscopic renal cryoablation (LRC). After a mean follow-up of 16.4 months (range 1-54), there were no recurrences. In such cases, tumors can either be excised en-bloc or separately. En-bloc excision extended the WIT because of the greater degree of reconstruction required. Although an advanced technique, excising adjacent tumors can safely be performed by treating them as a single mass. In select cases, LRC was found to be a useful adjunct to LPN. This is especially true where the tumors are geographically distant on the kidney and the patient has compromised renal function or nephron mass, where minimizing ischemia to the kidney is of critical importance.

## HEMINEPHRECTOMY FOR LARGER TUMORS

There is evidence to suggest that elective LPN for tumors 4-7 cm (T1b) may be a reasonable option in selected (i.e. favorable tumor characteristics) and well-counseled patients.[8–10] Our group has compared the outcomes of laparoscopic heminephrectomy in 41 patients requiring a resection >30% of renal parenchyma to a contemporary group of 41 consecutive patients who underwent LPN with <30% resection.[39] Other than a longer WIT (39 vs. 33 min) for laparoscopic heminephrectomy, there were no differences between the two groups as regards EBL, OR time, analgesic requirement, hospital stay, postoperative serum Creatinine, and overall complications. All surgical margins were negative. Specific technical considerations for laparoscopic heminephrectomy include achieving adequate surgical margins, entry into the pelvicaliceal system requiring suture repair, transection of sizable deep renal sinus vessels, and securing durable renal hemostasis.

## CYSTIC LESIONS

We believe LPN for a cystic mass is technically more challenging due to the greater risk of inadvertent cyst rupture and subsequent tumor spillage. Our group compared 50 patients undergoing LPN for a cystic renal lesion to 50 consecutive patients undergoing LPN for a solid renal mass.[40] LPN was successful in all cases and intraoperative complications were similar in the two groups. All surgical margins were negative. However, 1 patient in the cyst group had retroperitoneal recurrence at 1 year despite negative margins. Avoiding direct contact between laparoscopic instruments and cyst wall is important in order to minimize chances of spillage.

## TUMORS ASSOCIATED WITH THE RENAL HILUM

Tumors located in the renal hilum and in contact with the main renal vessels and their extra-sinus branches have been considered by many groups to be a contraindication to LPN. In 2005, Gill *et al.* reported the outcomes of LPN for hilar tumors in 25 patients. Mean tumor size was 3.7cm (range 1-10.3).[41] LPN was successful in all cases without any open conversions or operative re-interventions. Hemorrhagic complications occurred in 3 patients (12%). Preoperative 3-D video reconstruction of triphasic spiral computerized tomography (CT) was invaluable in detailing the number, interrelationship, anatomical course and position of the renal vessels in relation to the tumor. Secure repair of the pelvicaliceal system was one of the greatest challenges of LPN for hilar tumors. Hilar branches from the main renal vasculature entering the tumor directly should be clipped securely. Careful dissection in the Gil-Vernet plane may allow some of the hilar vessels to be dissected away from the hilar tumor and thereby spared.

## TUMOR IN A SOLITARY KIDNEY

LPN for tumor in a solitary kidney is a technically and psychologically challenging operation. The margin for error is small and a mishap could render the patient anephric or on dialysis. There is a need to suture precisely and fast, while under pressure to keep clamp time to a minimum. Care should also be taken when clamping the renal hilum to avoid too much compression on the renal artery which could lead to an intimal tear. If the patient has a plaque at the ostium of the renal artery, the clamp should be applied more distally on the artery in order to minimize the risk of plaque rupture and embolism.

Gill *et al.* reported 22 patients of LPN for tumor in a solitary kidney.[42] Mean WIT was 29 minutes (range 14-55). Two cases (9%) were electively converted to open surgery. Median preoperative and postoperative serum creatinine (1.2 and 1.5 mg/dl) and estimated glomerular filtration rate (67.5 and 50 ml/min/1.73 m^2^) reflected a change of 33% and 27%, respectively. Important technical caveats include adequate intravenous hydration, reno-pharmacological protection with mannitol and furosemide, minimal ischemic insult and refined technique. If the kidney does not re-vascularize after unclamping, as demonstrated by restoration of color and turgor, topical papaverine through a long laparoscopic needle should be used. If this does not produce the desired effect within a few minutes, intra-operative Doppler ultrasound should be performed to check for flow in the main renal artery and vein as well as in the arcuate vessels in the renal parenchyma.

## CENTRALLY LOCATED TUMORS

Centrally located tumors typically require precise intracorporeal suturing and complex reconstruction in a time-sensitive manner. The technical complexity of such cases depends on where the central tumor is located and what kind of suturing angles are available, especially as regards the dominant hand. Frank *et al.* compared LPN for central tumors (n=154) with LPN for peripheral tumors (n=209).[43] Central tumors were defined as those abutting or invading the collecting system on preoperative CT. Although EBL was similar, central tumors required longer OR time, WIT and hospital stay. There was one positive margin in each group. However, there were more early postoperative complications in the central group.

Robotic surgery may be particularly helpful for central tumors as it allows magnified 3-D visualization of the collecting system as well as intra-sinus blood vessels. In addition, the issues germane to good suturing angles in straight LPN are not as critical with robotic LPN, given the wristed robotic needle drivers.

## LPN AND INCIDENTAL ≥ PT2 TUMORS

Ukimura *et al.* reported results of LPN in patients with an incidentally detected stage pT2 (n=1), pT3a (n=19) and pT3b (n=1) tumor in 21 patients.[44] Neither the preoperative CT scan nor intraoperative US could definitively detect ≥ pT2 tumors. All resection margins were negative for cancer. In the one case with stage T3b, detection of the tumor invasion of a renal vein branch, resulted in conversion to LRN. During a mean followup of 29 months (range 1-58), the cancer-specific survival was 95%. In order to avoid a positive margin, and a potential local recurrence, it is of critical importance to routinely excise the overlying fat en bloc with the tumor.

## LPN IN OBESE PATIENTS

Obesity is associated with an increased risk of RCC and surgical management of obese patients is associated with a greater risk of intraoperative and postoperative complications.[45–46] Therefore, until recently obesity was considered a relative contraindication for LPN. Our group has compared the operative data and postoperative complications of 140 obese (BMI >30 kg/m^2^) and 238 nonobese (BMI ≤30 kg/m^2^) patients.[47] LPN was performed safely in obese patients with a perioperative complication rate similar to that of nonobese patients. The retroperitoneal approach was associated with a shorter OR time and hospital stay in both groups. Port placement needs to be modified for morbidly obese patients. In general, the ports are placed more cephalad and laterally. Bariatric ports and instruments may be necessary. These patients also have substantial vessels in the perinephric fat which need to be controlled while defatting the kidney.

## LPN AFTER PREVIOUS IPSILATERAL RENAL SURGERY

Prior ipsilateral renal surgery is considered a relative contraindication for LPN, because of the likelihood of dense adhesions and distorted tissue planes. We have performed 25 LPN (16 transperitoneal, 9 retroperitoneal) after previous ipsilateral renal surgery (unpublished data). Mean tumor size was 2.5 cm (range 1-5.6), interval from previous surgery was 6.6 years (range 0.3-34) and WIT was 35.8 minutes (range 22-57).[48] We believe LPN can be technically challenging after prior surgery and adequate experience is necessary for good outcomes. Careful planning of port placement, meticulous dissection around the hilum, and establishing a standard protocol during surgery are the main considerations for successful LPN after prior surgery. Image fusion technologies may be useful in the future to allow ready identification of hilar vessels, the tumor, and other landmarks, even in the presence of substantial adhesions.

## ONCOLOGIC OUTCOMES

LPN is increasingly becoming a definitive therapeutic option in patients with small renal mass. However, optimizing the oncologic efficacy of a given cancer operation is of paramount importance. Lane and Gill have recently published the initial report of oncological outcomes 5 years after LPN.[12] At a median followup of 5.7 years there was only one local recurrence and no distant metastasis. No patient with normal serum creatinine undergoing elective LPN developed chronic renal insufficiency. Overall and cancer specific survival was 86% and 100%, respectively.

## FUTURE PROSPECTS

In order to further expand indications for LPN as well as disseminate the technique to more centers the technical complexity of the procedure needs to decrease, and a reliable method of decreasing or eliminating warm ischemia needs to emerge. As of this writing there is no foolproof alternative to advanced laparoscopic suturing to achieve reliable hemostasis and PCS closure. Robotics will undoubtedly help those who are hesitant about laparoscopic suturing.[49–50] Unclamped LPN is not a reality today, except for the most superficial lesions. Novel hemostatic-urinary sealants could make this possible in the future, especially for peripheral lesions. Whether laser excision or water-jet dissection could make a difference in the future remains to be seen. As of now, neither are major players in the LPN arena. Advances in intra-operative imaging and surgical navigation are needed to guide the tumor excision in real-time in order to minimize chances of a positive margin while maximizing nephron preservation.

## CONCLUSIONS

LPN offers perioperative and oncologic outcomes comparable to OPN, while decreasing morbidity associated with a flank incision. In experienced hands, complex tumors such as large, cystic, hilar, central, multiple, intraparenchymal and ≥ pT2 tumors can be effectively and safely treated with LPN. Given the requisite expertise and experience, tumor in a solitary kidney, concomitant en bloc adrenalectomy, the presence of renal artery disease, tumor in a horseshoe kidney, obesity, and prior ipsilateral renal surgery are no longer contraindications for LPN. Robotics is a welcome extension to the art and science of LPN and is likely to bring it within reach of more urologists who have not yet embraced LPN due to the complex nature of time-sensitive laparoscopic suturing in the partial nephrectomy bed. In 2009, LPN is well suited for the majority of renal tumors, while OPN should be reserved for the most complex tumors.

## References

[1] Lerner SE, Hawkins CA, Blute ML, Grabner A, Wollan PC, Eickholt JT (2002). Disease outcome in patients with low stage renal cell carcinoma treated with nephron sparing or radical surgery. J Urol.

[2] Lee CT, Katz J, Shi W, Thaler HT, Reuter VE, Russo P (2000). Surgical management of renal tumors 4 cm or less in a contemporary cohort. J Urol.

[3] McKiernan J, Simmons R, Katz J, Russo P (2002). Natural history of chronic renal insufficiency after partial and radical nephrectomy. Urology.

[4] Kutikov A, Fossett LK, Ramchandani P, Tomaszewski JE, Siegelman ES, Banner MP (2006). Incidence of benign pathologic findings at partial nephrectomy for solitary renal mass presumed to be renal cell carcinoma on preoperative imaging. Urology.

[5] Belldegrun A, Tsui KH, deKernion JB, Smith RB (1999). Efficacy of nephron-sparing surgery for renal cell carcinoma: analysis based on the new 1997 tumor-node-metastasis staging system. J Clin Oncol.

[6] Lau WK, Blute ML, Weaver AL, Torres VE, Zincke H (2000). Matched comparison of radical nephrectomy vs nephron-sparing surgery in patients with unilateral renal cell carcinoma and a normal contralateral kidney. Mayo Clin Proc.

[7] Huang WC, Levey AS, Serio AM, Snyder M, Vickers AJ, Raj GV (2006). Chronic kidney disease after nephrectomy in patients with renal cortical tumours: a retrospective cohort study. Lancet Oncol.

[8] Patard JJ, Shvarts O, Lam JS, Pantuck AJ, Kim HL, Ficarra V (2004). Safety and efficacy of partial nephrectomy for all T1 tumors based on an international multicenter experience. J Urol.

[9] Leibovich BC, Blute ML, Cheville JC, Lohse CM, Weaver AL, Zincke H (2004). Nephron sparing surgery for appropriately selected renal cell carcinoma between 4 and 7 cm results in outcome similar to radical nephrectomy. J Urol.

[10] Becker F, Siemer S, Hack M, Humke U, Ziegler M, Stöckle M (2006). Excellent long-term cancer control with elective nephron-sparing surgery for selected renal cell carcinomas measuring more than 4 cm. Eur Urol.

[11] Gill IS, Desai MM, Kaouk JH, Meraney AM, Murphy DP, Sung GT (2002). Laparoscopic partial nephrectomy for renal tumor: duplicating open surgical techniques. J Urol.

[12] Lane BR, Gill IS (2007). 5-Year outcomes of laparoscopic partial nephrectomy. J Urol.

[13] Gill IS, Ramani AP, Spaliviero M, Xu M, Finelli A, Kaouk JH (2005). Improved hemostasis during laparoscopic partial nephrectomy using gelatin matrix thrombin sealant. Urology.

[14] Aron M, Gill IS (2007). Minimally Invasive Nephron-Sparing Surgery (MINSS) for Renal Tumours Part I: Laparoscopic Partial Nephrectomy. Eur Urol.

[15] Winfield HN, Donovan JF, Godet AS, Clayman RV (1993). Laparoscopic partial nephrectomy: initial case report for benign disease. J Endourol.

[16] Gill IS, Delworth MG, Munch LC (1994). Laparoscopic retroperitoneal partial nephrectomy. J Urol.

[17] McDougall EM, Elbahnasy AM, Clayman RV (1998). Laparoscopic wedge resection and partial nephrectomy-the Washington University experience and review of the literature. JSLS.

[18] Janetschek G, Jeschke K, Peschel R, Strohmeyer D, Henning K, Bartsch G (2000). Laparoscopic surgery for stage T1 renal cell carcinoma: radical nephrectomy and wedge resection. Eur Urol.

[19] Wright JL, Porter JR (2005). Laparoscopic partial nephrectomy: comparison of transperitoneal and retroperitoneal approaches. J Urol.

[20] Ng CS, Gill IS, Ramani AP, Steinberg AP, Spaliviero M, Abreu SC (2005). Transperitoneal versus retroperitoneal laparoscopic partial nephrectomy: patient selection and perioperative outcomes. J Urol.

[21] Herrell SD, Levin BM (2005). Laparoscopic partial nephrectomy: use of TissueLink hemostatic dissection device. J Endourol.

[22] Moinzadeh A, Gill IS, Rubenstein M, Ukimura O, Aron M, Spaliviero M (2005). Potassium-titanyl-phosphate laser laparoscopic partial nephrectomy without hilar clamping in the survival calf model. J Urol.

[23] Lotan Y, Gettman MT, Lindberg G, Napper CA, Hoopman J, Pearle MS (2004). Laparoscopic partial nephrectomy using holmium laser in a porcine model. JSLS.

[24] Moinzadeh A, Hasan W, Spaliviero M, Finelli A, Kilciler M, Magi-Galluzzi C (2005). Water jet assisted laparoscopic partial nephrectomy without hilar clamping in the calf model. J Urol.

[25] Guillonneau B, Bermúdez H, Gholami S, El Fettouh H, Gupta R, Adorno Rosa J (2003). Laparoscopic partial nephrectomy for renal tumor: single center experience comparing clamping and no clamping techniques of the renal vasculature. J Urol.

[26] Shekarriz B, Shah G, Upadhyay J (2004). Impact of temporary hilar clamping during laparoscopic partial nephrectomy on postoperative renal function: a prospective study. J Urol.

[27] Thompson RH, Frank I, Lohse CM, Saad IR, Fergany A, Zincke H (2007). The impact of ischemia time during open nephron sparing surgery on solitary kidneys: a multi-institutional study. J Urol.

[28] Nguyen MM, Gill IS (2008). Halving ischemia time during laparoscopic partial nephrectomy. J Urol.

[29] Gill IS, Abreu SC, Desai MM, Steinberg AP, Ramani AP, Ng C (2003). Laparoscopic ice slush renal hypothermia for partial nephrectomy: the initial experience. J Urol.

[30] Landman J, Venkatesh R, Lee D, Vanlangendonck R, Morissey K, Andriole GL (2003). Renal hypothermia achieved by retrograde endoscopic cold saline perfusion: technique and initial clinical application. Urology.

[31] Janetschek G, Abdelmaksoud A, Bagheri F, Al-Zahrani H, Leeb K, Gschwendtner M (2004). Laparoscopic partial nephrectomy in cold ischemia: renal artery perfusion. J Urol.

[32] Desai MM, Gill IS, Kaouk JH, Matin SF, Novick AC (2003). Laparoscopic partial nephrectomy with suture repair of the pelvicaliceal system. Urology.

[33] Fazio LM, Downey D, Nguan CY, Karnik V, Al-Omar M, Kwan K (2006). Intraoperative laparoscopic renal ultrasonography: use in advanced laparoscopic renal surgery. Urology.

[34] Turna B, Aron M, Gill IS (2008). Expanding indications for laparoscopic partial nephrectomy. Urology.

[35] Ramani AP, Abreu SC, Desai MM, Steinberg AP, Ng C, Lin CH (2003). Laparoscopic upper pole partial nephrectomy with concomitant en bloc adrenalectomy. Urology.

[36] Steinberg AP, Abreu SC, Desai MM, Ramani AP, Kaouk JH, Gill IS (2003). Laparoscopic nephron-sparing surgery in the presence of renal artery disease. Urology.

[37] Molina WR, Gill IS (2003). Laparoscopic partial nephrectomy in a horseshoe kidney. J Endourol.

[38] Steinberg AP, Kilciler M, Abreu SC, Ramani AP, Ng C, Desai MM, Kaouk JH (2004). Laparoscopic nephron-sparing surgery for two or more ipsilateral renal tumors. Urology.

[39] Finelli A, Gill IS, Desai MM, Tan YH, Moinzadeh A, Singh D (2005). Laparoscopic heminephrectomy for tumor. Urology.

[40] Spaliviero M, Herts BR, Magi-Galluzzi C, Xu M, Desai MM (2005). Laparoscopic partial nephrectomy for cystic masses. J Urol.

[41] Gill IS, Colombo JR, Frank I, Moinzadeh A, Kaouk J, Desai M (2005). Laparoscopic partial nephrectomy for hilar tumors. J Urol.

[42] Gill IS, Colombo JR, Moinzadeh A, Finelli A, Ukimura O, Tucker K (2006). Laparoscopic partial nephrectomy in solitary kidney. J Urol.

[43] Frank I, Colombo JR, Rubinstein M, Desai M, Kaouk J, Gill IS (2006). Laparoscopic partial nephrectomy for centrally located renal tumors. J Urol.

[44] Ukimura O, Haber GP, Remer EM, Gill IS (2006). Laparoscopic partial nephrectomy for incidental stage pT2 or worse tumors. Urology.

[45] Chow WH, Gridley G, Fraumeni JF, Järvholm B (2000). Obesity, hypertension, and the risk of kidney cancer in men. N Engl J Med.

[46] Mendoza D, Newman RC, Albala D, Cohen MS, Tewari A, Lingeman J (1996). Laparoscopic complications in markedly obese urologic patients (a multi-institutional review). Urology.

[47] Colombo JR, Haber GP, Aron M, Xu M, Gill IS (2007). Laparoscopic partial nephrectomy in obese patients. Urology.

[48] Turna B, Aron M, Frota R, Desai MM, Kaouk J, Gill IS (2008). Feasibility of laparoscopic partial nephrectomy after previous ipsilateral renal procedures. Urology.

[49] Benway BM, Bhayani SB, Rogers CG, Dulabon LM, Patel MN, Lipkin M (2009). Robot assisted partial nephrectomy versus laparoscopic partial nephrectomy for renal tumors: a multi-institutional analysis of perioperative outcomes. J Urol.

[50] Gautam G, Benway BM, Bhayani SB, Zorn KC (2009). Robot-assisted partial nephrectomy: current perspectives and future prospects. Urology.

